# Genome evolution of dengue virus serotype 1 under selection by *Wolbachia pipientis* in *Aedes aegypti* mosquitoes

**DOI:** 10.1093/ve/vead016

**Published:** 2023-03-03

**Authors:** Duong Thi Hue Kien, Kathryn Edenborough, Daniela da Silva Goncalves, Tran Thuy Vi, Etiene Casagrande, Huynh Thi Le Duyen, Vo Thi Long, Le Thi Dui, Vu Thi Tuyet Nhu, Nguyen Thi Giang, Huynh Thi Xuan Trang, Elvina Lee, I’ah Donovan-Banfield, Huynh Thi Thuy Van, Nguyen Minh Nguyet, Nguyen Thanh Phong, Nguyen Van Vinh Chau, Bridget Wills, Sophie Yacoub, Heather Flores, Cameron Simmons

**Affiliations:** World Mosquito Program, Institute of Vector-Borne Disease, Monash University, Clayton, VIC 3800, Australia; Department of Microbiology, Biomedicine Discovery Institute, Monash University, Clayton, VIC 3800, Australia; Oxford University Clinical Research Unit, Hospital for Tropical Disease, Ho Chi Minh City, Vietnam; World Mosquito Program, Institute of Vector-Borne Disease, Monash University, Clayton, VIC 3800, Australia; Oxford University Clinical Research Unit, Hospital for Tropical Disease, Ho Chi Minh City, Vietnam; World Mosquito Program, Institute of Vector-Borne Disease, Monash University, Clayton, VIC 3800, Australia; School of Biological Sciences, Monash University, Clayton, VIC 3800, Australia; Oxford University Clinical Research Unit, Hospital for Tropical Disease, Ho Chi Minh City, Vietnam; Oxford University Clinical Research Unit, Hospital for Tropical Disease, Ho Chi Minh City, Vietnam; Oxford University Clinical Research Unit, Hospital for Tropical Disease, Ho Chi Minh City, Vietnam; Oxford University Clinical Research Unit, Hospital for Tropical Disease, Ho Chi Minh City, Vietnam; Oxford University Clinical Research Unit, Hospital for Tropical Disease, Ho Chi Minh City, Vietnam; Oxford University Clinical Research Unit, Hospital for Tropical Disease, Ho Chi Minh City, Vietnam; World Mosquito Program, Institute of Vector-Borne Disease, Monash University, Clayton, VIC 3800, Australia; World Mosquito Program, Institute of Vector-Borne Disease, Monash University, Clayton, VIC 3800, Australia; Oxford University Clinical Research Unit, Hospital for Tropical Disease, Ho Chi Minh City, Vietnam; Hospital for Tropical Diseases, 190 Ben Ham Tu, District 5, Ho Chi Minh City, Vietnam; Hospital for Tropical Diseases, 190 Ben Ham Tu, District 5, Ho Chi Minh City, Vietnam; Oxford University Clinical Research Unit, Hospital for Tropical Disease, Ho Chi Minh City, Vietnam; Nuffield Department of Medicine, University of Oxford, Oxford, UK; Oxford University Clinical Research Unit, Hospital for Tropical Disease, Ho Chi Minh City, Vietnam; World Mosquito Program, Institute of Vector-Borne Disease, Monash University, Clayton, VIC 3800, Australia; School of Biological Sciences, Monash University, Clayton, VIC 3800, Australia; World Mosquito Program, Institute of Vector-Borne Disease, Monash University, Clayton, VIC 3800, Australia; Department of Microbiology, Biomedicine Discovery Institute, Monash University, Clayton, VIC 3800, Australia; Oxford University Clinical Research Unit, Hospital for Tropical Disease, Ho Chi Minh City, Vietnam

**Keywords:** dengue virus, *w*Mel *Wolbachia*, virus evolution, *Aedes aegypti* mosquitoes, biocontrol

## Abstract

The introgression of antiviral strains of *Wolbachia* into *Aedes aegypti* mosquito populations is a public health intervention for the control of dengue. Plausibly, dengue virus (DENV) could evolve to bypass the antiviral effects of *Wolbachia* and undermine this approach. Here, we established a serial-passage system to investigate the evolution of DENV in *Ae. aegypti* mosquitoes infected with the *w*Mel strain of *Wolbachia*. Using this system, we report on virus genetic outcomes after twenty passages of serotype 1 of DENV (DENV-1). An amino acid substitution, E203K, in the DENV-1 envelope protein was more frequently detected in the consensus sequence of virus populations passaged in *w*Mel-infected *Ae. aegypti* than wild-type counterparts. Positive selection at residue 203 was reproducible; it occurred in passaged virus populations from independent DENV-1-infected patients and also in a second, independent experimental system. In wild-type mosquitoes and human cells, the 203K variant was rapidly replaced by the progenitor sequence. These findings provide proof of concept that *w*Mel-associated selection of virus populations can occur in experimental conditions. Field-based studies are needed to explore whether *w*Mel imparts selective pressure on DENV evolution in locations where *w*Mel is established.

## Introduction

Dengue is a public health burden in many tropical countries and is responsible for significant morbidity and health-care expenditure (Martelli et al. 2015; Stanaway et al. 2016; Wilastonegoro et al. 2020). The deployment of *Wolbachia*-infected mosquitoes is one evidence-based, public health approach that locally controls dengue (O’Neill et al. 2018; Nazni et al. 2019; Ryan et al. 2019; Indriani et al. 2020; Pinto et al. 2021; Utarini et al. 2021). Developed and implemented by the World Mosquito Program, the replacement approach has established *Wolbachia* in *Aedes aegypti* populations within cities and towns in regions with high dengue incidence: Asia, Latin America, and Oceania.

The approach harnesses the collective host effects of *Wolbachia*, an endosymbiont that can rapidly introgress into mosquito populations by virtue of maternal transmission and cytoplasmic incompatibility (Sinkins 2004; Xi, Khoo, and Dobson 2005; Walker et al. 2011; Ross et al. 2017). The main *Wolbachia* strain deployed in the field (*w*Mel) confers protection to *Ae. aegypti*—and indirectly protects humans—from medically important arboviruses, including all four serotypes of dengue virus (DENV1-4) (Moreira et al. 2009; Walker et al. 2011; Frentiu et al. 2014; Fraser et al. 2017; Carrington et al. 2018). DENV-1 is marginally less inhibited by *w*Mel than the other three serotypes (Ferguson et al. 2015; Carrington et al. 2018; Flores et al. 2020). Plausibly, incomplete viral inhibition might spawn variants, accelerating virus evolution.

The lifespan of the approach might hinge on the stability of the tripartite relationship between *Ae. aegypti, Wolbachia,* and DENV. Interestingly, the *w*Mel genome is highly stable when introgressed into *Ae. aegypti* populations, with limited evolution after several years in the field (Huang et al. 2020; Dainty et al. 2021; Ross et al. 2022). In contrast, DENV1-4 are positive-sense, single-stranded ribonucleic acid (RNA) viruses with naturally mutable genomes attested by the divergence of multiple viral lineages within each serotype (Holmes and Twiddy 2003; Twiddy, Holmes, and Rambaut 2003).

One inherent risk then, to the success of the *Wolbachia* introgression approach, is the emergence of DENV variants that escape the virus-specific inhibition in *w*Mel-infected mosquitoes. Escape variants might be selected if they bypass the multifactorial intracellular modifications induced by *Wolbachia* infection, such as changes to lipid content and alteration of the cytoskeleton and endoplasmic reticulum membranes (Edenborough et al. 2021; Geoghegan et al. 2017; A. Lindsey et al. 2018). These organelles are critical for DENV replication (Samsa et al. 2009; Heaton and Randall 2010; Perera et al. 2012; Junjhon et al. 2014), such that the mutational requirements for escape might lead to viral fitness trade-offs. Unfit DENV variants are likely to be removed from the virus population via purifying selection (Holmes 2003) and might be thus challenging to identify directly in the field.

Understanding if *w*Mel can exert selective pressure on the DENV genome under experimental conditions might help to identify escape variants and facilitate surveillance in the field. Thus, this study compared the viral genetic variants that evolved during the serial passage of DENV-1 in *w*Mel-infected versus wild-type (WT) *Ae. aegypti*. With this system, *w*Mel-induced positive selection of amino acid residues in the DENV-1 envelope (E) was observed.

## Results

### Adaptive evolution of DENV-1 in *w*Mel-mosquitoes features a positive selection of codons in the DENV-1 envelope

We developed an *in vivo* DENV serial-passage system and used it to study virus evolution in the presence of *w*Mel. The system involved oral-feeding cohorts of WT and *w*Mel-infected *Ae. aegypti* with viraemic blood from DENV-1 (genotype I)-infected adult dengue patients, in the Hospital for Tropical Diseases in Ho Chi Minh City (HCM), Vietnam.

**Figure1.**
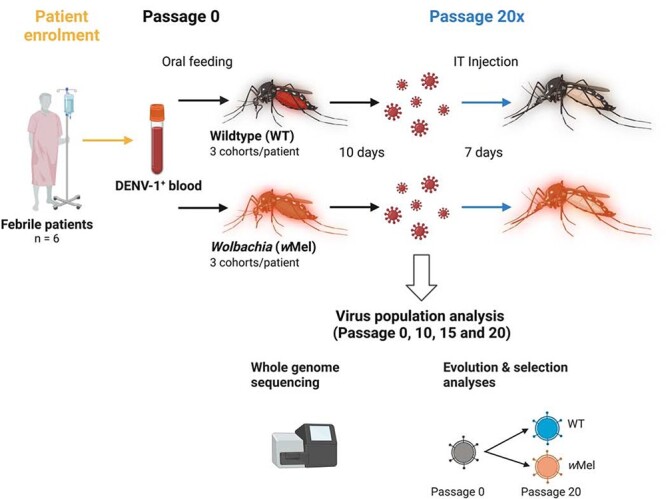
The schematic of DENV-1 passage system in WT- and *w*Mel-infected *Ae. aegypti*. Six febrile dengue patients were enrolled in the study, and their blood samples were fed to *Ae. aegypti* that were either WT (WT-mosquitoes) or infected with *Wolbachia* (*w*Mel-mosquitoes). Blood feeding was used to establish DENV infection for the first passage (passage 0). Ten days after blood feeding, *w*Mel- and WT-mosquitoes were collected and processed to produce a virus-containing supernatant. For each patient sample and mosquito line, supernatants from twenty mosquitoes were combined to form three independent cohorts. The three cohorts were re-inoculation into the cognate mosquito line via IT injection, and after 7 days, mosquitoes were collected and processed to produce virus-containing supernatant for the first passage (passage 1). The IT injection and collection process were repeated to passage 20. Supernatants from the three independent cohorts were processed separately and injected with fresh needles for each cohort to retain three independent replicates. Viral RNA extracted at passages 0, 10, 15, and 20 was subjected to qPCR and whole-genome sequencing. This figure was created with BioRender.com.

A total of six acute viraemic blood samples from six independent dengue patients established eighteen cohorts of WT-mosquitoes (three replicate cohorts per patient) and eighteen cohorts of *w*Mel-infected *Ae. aegypti* (three replicate cohorts per patient) with an HCM genetic background. Ten days after blood feeding, the virus populations in each cohort of mosquitoes were serially passaged twenty times, via intrathoracic (IT) injection with a 7-day incubation period, through their cognate mosquito line. The schematic in Fig. 1 represents the serial-passage system. Using a large volume of virus for passaging resulted in comparable viral genome copies between mosquito lines (*w*Mel and WT) for all patients and passages (P0–P20), and genome copies were sufficient for whole-genome sequencing (Fig. S1). *w*Mel density in the mosquito cohorts was also tested and remained stable between passage numbers (Fig. S2).

**Figure 2.**
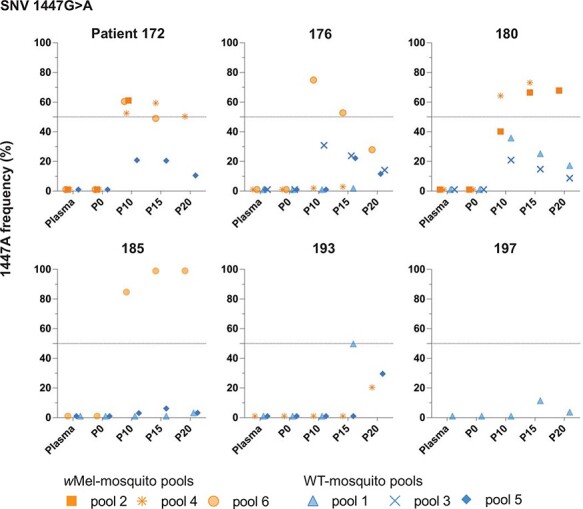
The evolution of the 1447G>A DENV-1 genomic variant in wMel- and WT-mosquitoes. DENV-1 derived from patient blood was passaged twenty times in wMel- and WT-mosquitoes. One SNV, 1447G>A (E203K), increased in frequency between P0 and P20 and resulted in a consensus change of the DENV-1 genome (>50 per cent frequency). The SNV frequency (%) of the 1447G>A SNV in plasma and in wMel- (orange square, star and circle symbols) or WT- (blue triangle, X mark and diamond symbols) mosquito cohorts at P0, 10, 15, and 20 is shown per patient. The frequency is shown for individual mosquito cohorts by unique symbols. Cohorts negative for the 1447G>A SNV over the entire duration of passage are not shown.

**Figure 3.**
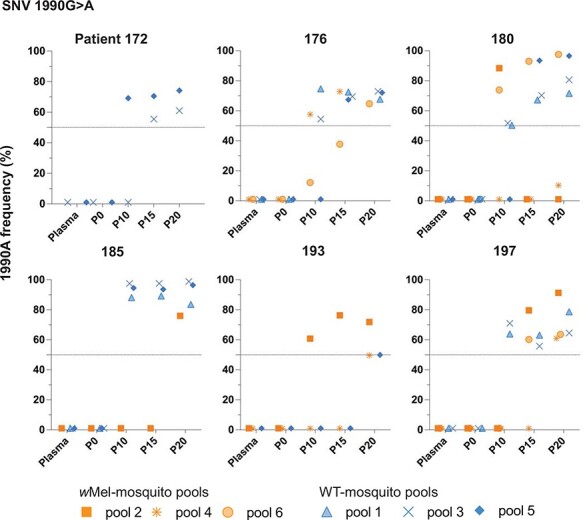
The evolution of the 1990G>A DENV-1 genomic variant in wMel- and WT-mosquitoes. The 1990G>A (E384K) SNV resulted in a consensus change of the DENV-1 genome and increased in frequency between P0 and P20 in both mosquito lines. The SNV frequency (%) of 1990G>A SNV in plasma and wMel- (orange square, star and circle symbols) or WT- (blue triangle, X mark and diamond symbols) mosquito cohorts at P0, 10, 15, and 20 is shown per patient. The frequency is shown for individual mosquito cohorts by unique symbols. Cohorts negative for the 1990G>A SNV over the entire duration of passage are not shown.

We hypothesized that virus single-nucleotide variants (SNVs) providing a selective advantage in the presence of *w*Mel would increase in frequency over passage in *w*Mel and not WT-mosquito cohorts and ultimately appear in the consensus sequence (>50 per cent frequency) (Lequime et al. 2017). By P20, two SNVs had met our predefined temporal and 50 per cent prevalence thresholds, i.e. present in more than half of the total mapped reads in a cohort and increased temporally between P0 and P20. The two SNVs were located at 1447 and 1990 in the envelope (E) coding region. The 1447G>A SNV was detected at >50 per cent frequency in seven of eighteen *w*Mel-mosquito cohorts originating from four of the total six independent patient plasma samples (Fig. 2 and Table S1). In contrast, 1447G>A was not detected above 50 per cent in any WT-mosquito cohort at any passage number from any patient plasma sample. When observed in WT-mosquitoes, 1447G>A was detected only transiently and at low frequency. The negative and positive controls included in our passage system and sequencing pipeline suggested that low-level detection of 1447A in WT-mosquitoes was unlikely to result from cross-sample contamination (Table S5); however, we cannot exclude this possibility altogether. The 1990G>A SNV was present at >50 per cent frequency in nine of eighteen *w*Mel- and thirteen of eighteen WT-mosquito cohorts in parallel across all six independent patient samples (Fig. 3 and Table S1).

SNVs 1447G>A and 1990G>A resulted in E➔K substitutions in the DENV envelope protein at amino acid residues 203 and 384 of the E protein, respectively. Over-representation of E203K in the *w*Mel-mosquito line might suggest that it is involved in the adaption to *w*Mel. The emergence of E384K in both mosquito lines suggests that this mutation might hold a general advantage for DENV-1 infection in mosquitoes. Interestingly, the 1447G>A and 1990G>A were not present with ≥50 per cent frequency in the same cohort of mosquitoes, raising the possibility of a fitness cost when both variants are present in a virus population.

### Stability of 1447A (203K) in mosquitoes in the presence and absence of *w*Mel selective pressure

**Figure 4.**
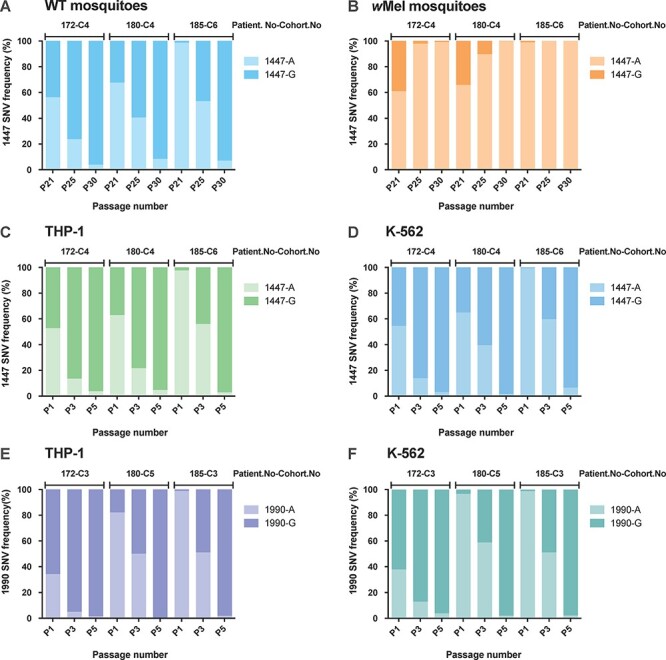
The decline in 1447A frequencies throughout the passage in WT-mosquitoes and human cell lines. *w*Mel-mosquito–adapted virus supernatants containing the 1447A variant at >50 per cent frequency were passaged 10x in WT (A) and *w*Mel-mosquitoes (B) and 5x in human cells lines: THP-1 (C) and K-562 (D). WT-mosquito–adapted virus supernatants containing 1990A at high frequencies were passaged 5x in THP-1 (E) and K-562 (F) cells. Bar charts show frequencies (% of reads containing each SNV of total reads) on the y-axis, which were determined by sequencing whole DENV genomes in passages 21, 25, and 30 mosquito cohorts and passages 1, 3, and 5 virus supernatants from human cells. SNV frequencies of the G form are represented by dark colours and the A form by light colours.

We hypothesized that the frequency of 1447A would decline in the absence of *w*Mel selection. To test this, we serially passaged P20 virus populations from *w*Mel-mosquito cohorts from patients 172, 180, and 185 ten times in WT and *w*Mel-mosquito cohorts. Each of these virus populations contained a >50 per cent frequency of the 1447A variant. We report on 1447A and G frequencies in each mosquito cohort at P21, P25, and P30 in Fig. 4 and Table S2. After one passage in WT-mosquitoes (P21), 1447A frequencies remained above 50 per cent consensus levels, yet these declined by P25, plummeting to <10 per cent in all three virus lineages by P30, with the 1447A variant being replaced by the WT progenitor sequence (1447G) (Fig. 4A).

The passage of the same three P20 supernatants revealed that 1447A persisted over ten passages in *w*Mel-infected mosquitoes; 1447A became fixed (>99 per cent) by P30 in all three lineages (Fig. 4B). These findings reinforced the concept of *w*Mel being the driving force underpinning 1447A (E203K) selection and suggest that 1447A was negatively selected in the absence of *w*Mel.

### Transience of 1447A (203K) and 1990A (384K) in human cell lines

DENV variants that are unfit or unstable in human cells might pose a reduced risk of establishing in the mosquito–human transmission cycle. We thus measured 203K and 384K variant frequency during DENV passage in human cells. In THP-1 and K-562 human cell lines, 1447A frequencies declined rapidly (<10 per cent) within five passages for all three of the virus lineages that were passaged. Reductions in 1447A were due to reversion to 1447G as the consensus within the virus population (Fig. 4C–D, Table S2). We also quantified DENV genome copies for whole virus populations (containing 1447A and 1447G) to ascertain the magnitude of DENV infection in human cells. Mean DENV genome copies of three *w*Mel-adapted virus lineages were equivalent to mean levels measured during the passage of three progenitor virus lineages, and overall, DENV copy numbers increased with passage number (Table S3). This finding indicated that variant fluctuation did not result from poor susceptibility of the cell lines to DENV-1 infection.

We next asked if the rapid frequency decline in human cells was a specific observation for 1447A or common to other variants uncovered in our passaging study. In human cells, we passaged three supernatants from P20 WT-mosquito lineages from patients 172, 180, and 185 that contained high frequencies of 1990A; the SNV selected in both *w*Mel- and WT-mosquito cohorts. Similar to 1447A, 1990A also declined in frequency to <10 per cent by P5 (Fig. 4E-F). These findings suggest that 1447A (203K) and 1990A (384K) are not sufficiently competitive to persist in the consensus sequence for multiple rounds of infection in human cells.

### *w*Mel selection is reproducible with a different DENV-1 strain

To assess the robustness of the findings related to 1447G>A and 1990G>A SNVs, we replicated the serial-passage study using a tissue culture–grown DENV-1 genotype I isolate (DV1/Vietnam/2008) instead of viraemic blood and a different mosquito line (Australian *w*Mel-infected *Ae. aegypti* and an uninfected control line, cured from *w*Mel infection with tetracycline).

We observed higher *w*Mel densities in Australian mosquitoes than in the HCM mosquitoes used previously (Fig. S2). Differences in density could result from differential processing of Australian mosquito supernatants, which were not filtered to retain as much virus as possible for passage. Potentially, these unfiltered supernatants from Australian mosquitoes contained more cellular debris, which may account for the increased *Wolbachia* density detected. Nevertheless, we observed DENV-1 genome copies in Australian and HCM mosquito cohorts that were similar (Figs. S1 and S3).

**Figure 5.**
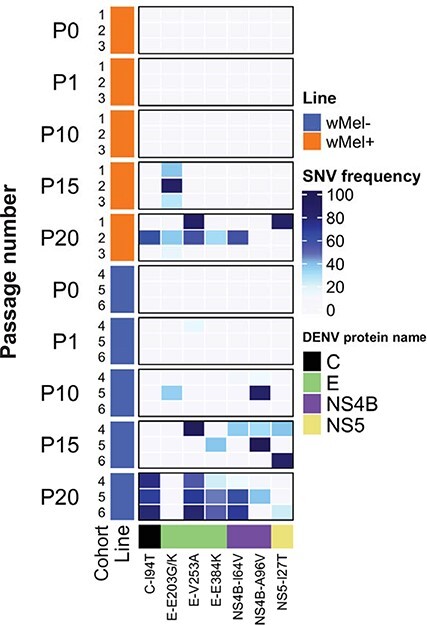
The heat map of non-synonymous substitutions that developed in the replicate DENV-1 passage experiment. DENV-1 was injected intrathoracically into mosquitoes to establish passage 0 (P0) in three mosquito cohorts per line. The three cohorts were passaged separately until P20 and DENV genomes sequenced to track evolution. SNV frequency is presented for non-synonymous substitutions only, showing only those that increased temporally with the passage. Amino acid substitutions within the specified DENV protein are shown on the x-axis for each passage number (P0, 1, 10, 15, and 20), mosquito cohort (1, 2, 3, 4, 5, and 6), and mosquito line (*w*Mel: orange and uninfected: blue). Each viral protein is annotated by colour (C: black, E: light green, NS4B: purple, and NS5: khaki). The heat map was produced with the ComplexHeatmap package in RStudio.

In support of our previous findings, the 1447G>A (E203K) and 1990G>A (E384K) SNVs developed *de novo* during the repeat serial-passage experiment. As we expected to observe these substitutions a priori, we report their frequency even when below the previously predefined 50 per cent threshold. The 1447G>A SNV was first identified at P15 in *w*Mel-mosquitoes at a mean frequency of 31.8 per cent ± 7.2 in two of the three mosquito cohorts and was identified again in one cohort at P20 (16.6 per cent) (Fig. 5 and Table S4). In the control mosquito line, 1447G>A arose in one P10 cohort at 34 per cent but was not present at P15 or P20. Sporadic emergence of 1447G>A in control mosquito lines is consistent with prior data (Fig. 2). At the same codon, a 1448A>G SNV (E203G) was detected in one *w*Mel-mosquito cohort at P15 (86.2 per cent) and P20 (35.0 per cent).

We identified an additional six non-synonymous substitutions that increased in frequency temporally. However, excluding one conservative substitution (A96V) in NS4B, all other substitutions were detected in both mosquito lines (Fig. 5). This included the 1990G>A (E384K) substitution in DENV-1, which was detected at P20 in one *w*Mel-mosquito cohort and all three control mosquito cohorts at a mean frequency of 40.3 per cent. The appearance of substitutions (E203K and E384K) in both experiments, although observed at lower frequencies in the second replicate experiment, highlights the overall reproducibility of these mutations in the context of a different mosquito genetic background and virus strain.

## Discussion

The likelihood of DENV evolving resistance to the antiviral potency of *w*Mel is an ill-defined risk to the *Wolbachia* introgression method. Here, we report DENV-1 genetic outcomes from a mosquito serial-passage system that identifies signals of *w*Mel-associated selection. The passage system defined amino acids in E under positive selection in *w*Mel-infected (203 and 384) and WT-mosquitoes (384).

The substitutions (E203K and E384K) arose independently in replicate cohorts during the passage of DENV that was acquired from individual DENV-1-positive patients. In a replicate experiment, where the DENV-1 strain and mosquito genetic background differed, both substitutions also arose within several technical replicate mosquito cohorts.

The recurring nature of 203 and 384 substitutions in this study might result from their centralized position within homopolymeric (HP) A tracts in the viral genome. HP tracts are sites at which RNA-dependent RNA polymerases readily induce mutations (Ratinier et al. 2008; Olspert, Carr, and Firth 2016).

Despite its recurrence, E203K was only weakly selected by *w*Mel in the replicate experiment. *w*Mel does not localize to all mosquito tissues, and its distribution can be focal (Amuzu et al. 2018; Fraser et al. 2020); hence, some somatic cells of the mosquito are likely to be *Wolbachia*-free. Interexperimental variation might be due to the stochastic nature of the variant and WT virus competition for replication in *Wolbachia*-free cells of the mosquito. Alternatively, E203K might impart fitness costs on different DENV-1 genetic backbones, therein limiting its selective advantage.

### Putative effects of E203K and E384K for DENV infection and *w*Mel blocking

DENV genome sequences encoding 203K are rarely observed (<5 per cent) in public viral genome repositories (92 per cent of 4100 sequences are 203E). As a consequence of low prevalence, the function of 203K in viral infection has not been studied for the DENV-1 serotype.

The E➔K substitution at residue 203, located in domain II of E, would increase local positive charge and position cationic side chains proximal to a conserved histidine (261H) based on the DENV-1 E 3D structure. Histidine–cation interactions are known as pH-sensing residues (Kudlacek et al. 2021) that trigger dimer-to-trimer rearrangement of E via electrostatic repulsion (Modis et al. 2003; Kampmann et al. 2006; Chaudhury, Ripoll, and Wallqvist 2015; Kuhn et al. 2015). We thus speculate that the 203 E➔K change, in the vicinity of 261H, might impact the kinetics of E trimerization and thus pH thresholds of fusion.

Hypothetically, the E203K substitution could modify fusion pH thresholds, such that the 203K variant might display atypical traversal of the endocytic pathway. We speculate that modified fusion thresholds could result in uncoating within early rather than late endosomes. Fusion with early endosomes might be beneficial for viral entry into *w*Mel-infected cells, as *w*Mel perturbs lipid homeostasis, causing defects in lipid processing within late endosomes (Geoghegan et al. 2017). Sufficient lipid concentration in late endosomes is crucial for DENV uncoating, and potentially, lipid dysregulation might inhibit this process (Zaitseva et al. 2010). It is important to recognize that the ability of the E203K variant to evade *w*Mel-induced virus blocking has not been studied here. This substitution might constitute the first step in the selection process, where *w*Mel-induced blocking is multifactorial and likely requires the accumulation of several mutations in the viral genome.

We speculate that the E384K variant, detected in WT- and *w*Mel-infected mosquitoes, may provide a general advantage for viral attachment to mosquito cells at 26–28°. Residue 384 is located within DIII of E, is highly surface exposed, and is part of a 10-amino acid loop that mediates binding to mosquito but not mammalian cells for DENV-2 (Hung et al. 2004). Four of these ten residues (382–385) are described in mosquito-borne but not tick-borne flaviviruses, suggesting that they may determine host tropism (Modis et al. 2003). Binding to mosquito cells in a constant temperature environment (26–28°) might select for E proteins with specific thermodynamic requirements, and limited exposure to human receptors might select for variants that bind mosquito receptors with high affinity.

### Virus passage studies and *Wolbachia*; limitations and relevance to the field

Key elements that set our passage system apart from prior studies (Koh et al. 2019; Martinez et al. 2019) include the use of oral feeding or a large IT injection volume to establish infection in mosquitoes. The delivery of small volumes is a standard practice for IT injection but might bottleneck genetic diversity in the virus population and likely contributed to *w*Mel-driven viral extinction in past passage studies in *Drosophila melanogaster* (Martinez et al. 2019). IT injection also bypasses midgut infection and escape barriers (Forrester et al. 2012), and its use might have altered the selective pressures in our passage system and the breadth of adaptive variants we thus observed. One difference though is we used large injection volumes to deliver high virus doses, which has been shown to limit the *w*Mel-mediated blockade of virus replication (Fraser et al. 2017). We link the use of a large bolus of viral inoculum, derived from whole mosquito bodies, to the interesting finding that typical *w*Mel-induced blocking of viral load was not observed.

Ultimately, we aim to determine the risk that 203K and 384K variants present to the *Wolbachia* method. Although both variants arise reproducibly in these passage experiments, they also readily revert to their progenitor forms (203E and 384E) in human cells and at least in the case of 203 in WT-mosquitoes. Transience of 203K and 384K in human cells might limit the risk of positive selection as DENV cycles between two hosts. Applying these results to the field, it is likely that 203K and 384K would be removed by virtue of purifying selection during mosquito-to-human transmission. A continued study of 203K and 384K variant fitness (i.e. kinetics of dissemination and transmission in mosquitoes), which we have not determined here, will provide more insight into the risk of these variants persisting in natural transmission cycles.

In summary, this study identified an amino acid change in the DENV-1 E protein that is reproducibly selected during the passage in *w*Mel-infected *Ae. aegypti*. The results demonstrate proof of concept that *w*Mel can impart selective pressure on the virus population in laboratory conditions. Whether such variants could evolve or are already present at low frequency in locations where *Wolbachia* has been introgressed into the *Ae. aegypti* population is unknown. Nonetheless, this laboratory-based detection of virus evolution could be used to inform prospective DENV population genetic studies in *Wolbachia* release sites.

## Materials and Methods

### Ethics Statement and Dengue Patient Cohorts

Mosquito colonies were blood-fed on adult, human volunteers in accordance with Monash University Human Research Ethics permit number CF11/0766-2011000387. Written informed consent was given by all volunteers before commencing. For blood feeding of mosquito colonies in Vietnam, adult volunteers provided written informed consent before their participation.

The study involving patient blood samples was carried out at the Oxford University Clinical Research Unit, HCM, Vietnam. It included feeding mosquitoes with viraemic blood collected from six febrile dengue patients. The relevant ethical protocols were reviewed and approved by the Scientific and Ethical Committee of the Hospital of Tropical Diseases (HTD) (EC CS/NÐ/16/27) and the Oxford Tropical Research Ethical Committee (OxTREC Reference: 45-16). Patients included in the study provided written informed consent and were ≥15 years of age, and inpatients at HTD with <96 h fever confirmed positive for DENV infection with an NS1 rapid test.

### Biosecurity and biosafety measures

At Monash University, mosquito infections were conducted within a quarantine insectary (AA-V2165) that operates at biosecurity containment level 3. The mutations identified in this serial-passage experiment are not known to increase pathogenicity and have been approved for recombinant virus production by the Office of the Gene Technology Regulator under the licence number DNIR-639.

### Virus serial-passage method

#### Derivation of mosquitoes and rearing

*Aedes aegypti* mosquitoes used in experiments were of either HCM or Australian (Cairns) genetic backgrounds. Australian *w*Mel and tetracycline-treated mosquito control lines were generated as previously described (Walker et al. 2011; Fraser et al. 2017). The HCM *w*Mel-mosquito line was produced by backcrossing Australian *w*Mel onto an HCM background and outcrossing the colonies as previously described (Carrington et al. 2018). All mosquitoes were maintained at 26–28°C, 65–85 per cent relative humidity (RH), and a 12:12 h light:dark cycle, with access to 10 per cent sucrose solution *ad libitum*. The HCM mosquitoes used in the experiments were from F_25_–F_46_ and G_28_–G_49_ for WT- and *w*Mel-mosquitoes, respectively. Australian mosquitoes used in the experiments were from G_37_–G_41_ and G_44_–G_48_ for *w*Mel and control lines, respectively.

#### Initial exposure of mosquitoes to viraemic patient blood

Patient blood samples were collected in ethylenediaminetetraacetic acid tubes and confirmed to contain DENV-1 with a serotype-specific quantitative polymerase chain reaction (qPCR) assay as previously described (Hue et al. 2011). Blood samples were fed to mosquitoes directly after collection to establish infection for passage 0. WT and *w*Mel females (2–4-day old) were orally fed for 30 minutes using artificial membrane feeders containing 300 µl of the patient’s blood. Mosquitoes were cold anaesthetized at 4°C for 45 seconds, and engorged females were transferred to 500 ml plastic containers. They were maintained in an environmental chamber at 27°C with 12:12 h light:dark cycle and 65–85 per cent RH.

#### Initial exposure of mosquitoes to tissue-culture–derived DENV

TVP/22776/Vietnam/2008 DENV-1 (GenBank accession FJ461335) was sourced from the World Reference Centre for Emerging Viruses and Arboviruses and amplified for two passages in C6/36 cells. Virus stocks were sequenced to confirm their identity and to generate a full-length genome for reference mapping. DENV-1 stock (1.2 × 10^6^ tissue culture infectious dose _50_/ml) was diluted 1:5 in Roswell Park Memorial Institute (RPMI) media and injected intrathoracically (69 nl) into mosquitoes to establish passage 0 mosquito cohorts.

#### Mosquito collection into cohorts

Seven to fourteen days post-exposure, mosquitoes were collected into RPMI media containing 2 per cent foetal calf serum (Gibco), 2 mM L-glutamine (Gibco), 2.5 µg/ml Fungizone, 50 U/ml penicillin, and 50 µg/ml streptomycin (Gibco). Mosquitoes were homogenized in media with a silica bead beater for 3 minutes at 30 Hz, and the supernatant was clarified by centrifugation for 1 minute, 1,400 relative centrifugal force (RCF), and passed through a 0.2-µm filter (Millipore). Supernatants (50 µl/mosquito) from twenty mosquitoes were combined to produce one cohort.

At each passage, triplicate cohorts were produced for each patient and mosquito line. Each cohort was maintained as an independent lineage over passage. For the passage experiment using tissue-culture–grown virus, mosquitoes were homogenized with a stainless-steel bead at 30 Hz for 1 minute with a TissueLyser II instrument (Qiagen). The supernatant was clarified via two centrifugation steps (5,000 RCF for 10 minutes at 4°C) rather than filtration.

#### Mosquito infection for serial passage

For passages 1–20, twenty naive mosquitoes were inoculated per cohort by IT injection with 0.5–1 µl of pooled supernatant. We performed twenty cycles of passaging of DENV in *w*Mel-infected or uninfected control *Ae. aegypti*. We also established negative (mosquitoes not injected with DENV-1) and positive (mosquitoes injected with a distinct DENV-1 strain Accession Number: FJ432735) controls to detect cross-sample contamination. The controls were run alongside test samples and assayed at passages 1, 5, 10, 15, and 20 according to the polymerase chain reaction (PCR) and sequencing pipelines outlined later.

### Virus and *Wolbachia* genome quantification

Viral RNA in patient blood and mosquito cohorts was extracted using either MagNA Pure 96 DNA and Viral NA Small Volume Kit (Roche) on the MagNA Pure 96 System or QIAamp viral RNA Kit (Qiagen) and quantified using previously described reverse transcription (RT)-qPCR assays (Hue et al. 2011; Fraser et al. 2017). The *Wolbachia* infection status in WT- and *w*Mel-infected females was confirmed by relative quantification of the *Wolbachia wsp* gene to the *Ae. aegypti rps17* reference gene as previously described (Fraser et al. 2017). All qPCR was performed using a LightCycler 480 II Instrument (Roche).

### Viral whole-genome sequencing

The extracted RNA was subjected to cDNA synthesis using SuperScript IV First-Strand Synthesis System (Thermo Fisher Scientific) according to the manufacturer instructions. Tiled amplification of the whole DENV genome was conducted in two separate multiplex PCR reactions with Q5 high-fidelity DNA polymerase (New England Biolabs) using previously described conditions (Quick et al. 2017).

The amplicons were purified with AMPure XP beads (Beckman Coulter) and pooled at equal concentrations, which were determined with the PicoGreen dsDNA quantitation assay (Beckman Coulter). Pooled DNA of 0.4 ng/μl was used as input for further library construction using Nextera XT DNA Library Preparation Kit (Illumina). Index samples were quantified with KAPA Library Quantification Kit (Roche) and then pooled together at 1.5–2 nM. The pooled library was sequenced with MiSeq v2 300 or v3 600 cycle reagent (Illumina). Passage samples and controls were pooled into six independent sequencing libraries, which were sequenced in separate runs as indicated by sequencing batch ID in Table S5.

### Next-generation sequencing analysis

Amplification and Illumina sequencing of DENV genomes was performed on triplicate cohorts of mosquitoes at P0, P10, P15, and P20. The total number of reads mapped to the reference sequence, coverage depth, and Q scores of viral genomes in passage material and controls are summarized in Tables S5 and S6.

#### Generating reference sequences for mapping

Sequence reads from the initial oral feed (passage 0) were assembled *de novo* with CLC Genomics Workbench 9.0 or Geneious Prime 2021.1.1. The longest contiguous sequence was searched in the nr/nt nucleotide collection of GenBank, in order to select the reference genome, which presented the highest homology. Sequence reads were then re-mapped to the homologous DENV-1 sequence to generate a full-length genome sequence suitable for reference-based mapping. For the passage experiment using tissue-culture–grown virus, reference sequences were generated by sequencing DENV-1 virus stocks.

#### Generating consensus sequences and SNV calling

Adapter trimming and fastq demultiplexing were performed with default Illumina pipelines. Further analyses were performed with CLC (for the patient-derived virus passage experiment) and a custom snakemake pipeline (for the tissue-culture–derived virus passage experiment). In CLC, read ends were trimmed based on a quality score limit of 0.05, and ambiguous (N) characters were removed with a limit of two allowed per read. Quality scores were calculated on a Phred scale and converted to base-calling error probability. Consensus sequences for the mosquito cohorts (passages 0, 10, 15, and 20) were achieved by mapping sequence reads to the passage 0 references for each cognate patient. The consensus sequence incorporates nucleotides with >10× of sequencing depth and variant frequency level >50 per cent.

To identify regions in read mappings with unexpectedly low or high coverage, the coverage analysis was run. The algorithm fits a Poisson distribution to the observed coverages in the positions of the mapping. The two parameters, ‘Minimum length’ and the ‘*P*-value threshold value’, were set at 50 and 0.0001, respectively.

Low-frequency variants were detected using a Neighbourhood Quality Standard model. For inclusion in the analysis, the minimum coverage cut-off was 1,000, and the per cent of minimum variant frequency cut-off was 0.5 per cent. Viral genomes in some mosquito cohorts did not meet the criteria of high coverage and were excluded from the study and included patient number 172: passages 15 (one *w*Mel-mosquito cohort) and 20 (two *w*Mel-mosquito cohorts); patient number 176: passages 10 (one *w*Mel-mosquito cohort), 15 (one *w*Mel-mosquito cohort), and 20 (two *w*Mel-mosquito cohorts); and patient number 193: passages 10 (one WT-mosquito cohort), 15 (one WT-mosquito cohort), and 20 (one WT-mosquito cohort).

For the tissue-culture–derived virus passage experiment, viral cDNA, tiled PCRs, and libraries were prepared in duplicate for each mosquito cohort. DENV primer sequences were removed from 5ʹ and 3ʹ read ends with Alien Trimmer (Criscuolo and Brisse 2013). Reads were also trimmed to remove low-quality bases (quality < 30) and small reads (length < 50 nucleotides). Trimmed reads were mapped to reference DENV genomes with bwa-mem (Li and Durbin 2009), and SNVs above a 10 per cent threshold were called with iVar (Grubaugh et al. 2019). Only variants that were present in duplicated libraries were included for further analysis. Sequence data are deposited at the National Center for Biotechnology Information under Accession Numbers MN912109.1–MN912248.1 and BioProject PRJNA898886.

### Human cell infection with DENV-1 variants

Five serial passages of each DENV-1 variant (either 203K or 384K in envelope) were conducted in THP-1 and K-562 cells. Cells (10^5^) were washed in RPMI with 2 per cent fetal bovine serum, exposed to 203K and 384K variants in a total volume of 0.2 ml, and incubated at 37°C for 2 hours with agitation every 20 minutes to prevent the cell sedimentation. Cells were then washed six times by centrifugation (900 RCF for 3 minutes) and resuspended in fresh media and finally seeded cells in 24-well plates for 7 days of incubation at 37°C with 5 per cent CO_2_. Supernatants were collected for four consecutive passages. The quantification of viral load by RT-qPCR and genome sequencing was performed at passages 1, 3, and 5.

### Statistical analyses

Analyses were conducted and plotted in RStudio (version 1.2.5033), R (version 3.4.4), and GraphPad Prism (version 8).

## Supplementary Material

vead016_Supp

## Data Availability

The raw data are available in the Supplementary Material.
